# Research on industrial structure adjustment and spillover effect in resource-based regions in the post-pandemic era

**DOI:** 10.1371/journal.pone.0296772

**Published:** 2024-01-19

**Authors:** Ziqiong He, Rongguang Zhang, Qiwen Qiu, Zhe Chen

**Affiliations:** 1 School of Management Science, Chengdu University of Technology, Chengdu, China; 2 College of Mathematics and Physics, Chengdu University of Technology, Chengdu, China; 3 International Research Centre of Big Data for Sustainable Development Goals(CBAS), Beijing, China; 4 Digital Hu Line Research Institute, Chengdu University of Technology, Chengdu, China; Chengdu University of Information Technology, CHINA

## Abstract

Resource-based regions support national economic development and are essential sources of basic energy and raw materials. In the post-pandemic era, however, there are practical situations to deal with, such as a fractured industrial chain, a weaker industrial structure, and a sharp reduction in economic benefits. Based on data collected from 68 cities in China, from 2010 to 2021, with 816 observations, this paper explores the industrial development process of resource-based regions in China and the change in the toughness of the industrial structure under the impact of COVID-19. The paper studies and analyzes industrial development trends, industrial structure toughness, and spatial spillover effects. The methods used are the Markov chain model and the Industrial Structure Advancement Index. By building the spatial Dubin model, the paper analyzes the spatial spillover effect of regional industrial development. It decomposes the spillover effect using the partial differential model based on regression. The results show that, during the study period, the comprehensive development level of industries in resource-based regions in China was slowly improving and tended to stabilize after entering the post-pandemic era. The evolution of an advanced industrial structure is significantly heterogeneous among regions, and each region has different toughness. The impact of COVID-19 has reduced the toughness of China’s resource-based regions’ industrial structure. The spatial spillover effect of regional industrial development is significant. Labor force, technology input, and industrial-structure optimization have different impacts on the industrial development of neighboring regions. In the post-pandemic era, China has used new management methods for more innovation. In order to achieve low-carbon, environmental protection, and sustainable development of resources, realize the rapid recovery of the toughness of industrial structure in China’s resource-based cities, and reduce the impact of the COVID-19 pandemic, China proposes to expand the supply of resources, improve the allocation of resources, optimize the direction, promote the rational flow and efficient aggregation of various factors, and enhance the impetus for innovation and development.

## 1. Introduction

Since the beginning of 2020, the global ferment caused by the outbreak of a novel coronavirus known as COVID-19 not only broke the existing order but also gradually changed industries in various regions [1]. In today’s China, one hundred years of change and the pandemic of the century are intertwined and superimposed, economic globalization is facing a counter-current, and the global industrial chain supply system is facing serious challenges [2, 3]. Based on the actual international and domestic environment, COVID-19 has repeatedly led to enterprise downtime and poor logistics and transportation [4, 5]. The industrial chain faces many "blocking points," and the toughness of the industrial structure has been significantly weakened [6]. Therefore, exploring the laws of industrial development and structural toughness in the post-pandemic era, ensuring the safety and stability of the industrial chain, and enhancing structural toughness have become major issues facing China [7, 8].

Since the 1980s, resource-based regions have developed rapidly, proving themselves a strong support for China’s economic development and an important strategic guarantee for the sustainable development of energy and resources [9]. These resource-based regions rely on the advantages of natural resources and play a vital role in securing the country’s early industrial sector and economic development [10, 11]. Since 2020, the whole country has entered the era of epidemic prevention and control. Affected by COVID-19, the rupture of the industrial chain in resource-based regions and the "suspension" of resource exploitation have further hindered economic development, making resource-based regions gradually become the most underdeveloped regions in China’s economic development [12–14]. What is needed is to study the industrial development of resource-based regions in the post-pandemic era, promote the adjustment of industrial structure, reduce the negative impact of COVID-19 on industry, reduce the dependence of industry on resources and the environment, and reshape the industrial chain and value chain [15–17]. These will not only help provide new ideas for the theoretical study of industrial structure adjustment in resource based-regions in China but also have guiding significance for the high-quality development of resource-based regions in China.

The Twentieth National Congress clearly instructed: "accelerate the construction of a new development pattern and strive to promote high-quality development." The development of resource-based regions is the top priority if China is to achieve regional coordination. After entering the post-pandemic era, the problems of resource-based regions—such as excessive dependence on factor input, industrial chain fracture, reduced industrial toughness, and irrational industrial structure—have become prominent [18]. Compared with existing research, the possible contributions of this paper are as follows: First, taking the COVID-19 outbreak as the turning point, the study empirically explores industrial development law, the toughness change in industrial structure, spatial layout characteristics, and economic input factor endowment in China’s resource-based regions. Secondly, the study measures the marginal production capacity of production factors in China’s resource-based regions in general and carries out exploratory analysis on the spatial spillover effect and influencing factors in China’s resource-based regions, using the spatial econometric model. Finally, The theoretical and empirical methods proposed or introduced in this paper provide research ideas, theoretical tools, analysis methods and countermeasures for solving problems such as improving the toughness of industrial structure, optimizing regional strategic layout and high-quality economic development in China’s resource-based regions in the post-pandemic era. They are also conducive to helping resource-based regions achieve the goals of low-carbon, environmental protection and sustainable development. In addition, after being affected by the COVID-19, self-analysis and evaluation and timely adjustment of the development direction have certain application value.

## 2. Literature review

The impact of epidemic diseases on the economy has always attracted the attention of the academic community. Due to the progress of modern medical and health technology, the threat of infectious diseases to human beings has been greatly reduced [19]. However, the economic cost of large-scale infectious diseases is huge, and researchers generally lack an understanding of the long-term impact of the pandemic. COVID-19 not only directly caused industrial economic losses, but its occurrence and spread were also is costly to prevent the occurrence and spread of the epidemic [20]. The global influenza pandemic of 1918–19 (known as the Spanish flu) killed 675,000 people in the United States, far more than the two World Wars, the Korean War, and the Vietnam War combined [21, 22]. Scholars have also conducted a wealth of studies on the impact of other epidemics on economic fluctuations. For example, the biggest economic loss caused by the influenza epidemic in the United States is the death of personnel and the production and consumption of goods directly affected by the shutdown [23]. H1NI influenza(swine flu) had a great impact on the macro-economy of Australia in a short period of time, especially on the service industries such as catering and tourism [24]. After the outbreak of a new coronavirus in 2020(COVID-19), some foreign scholars re-examined the impact of the Spanish flu epidemic on the US economy in order to provide a reference for the new coronavirus epidemic. One study found that the Spanish flu epidemic reduced GDP and consumption by an average of 6 to 8 percentage points [25].

As a major public health emergency, COVID-19 has had a significant impact on the economy and industrial development with many domestic and foreign scholars conducting empirical studies to assess the degree of impact. Abuzayed [26] used the ratio of new infection cases and death cases announced by the government as the proxy variable of epidemic impact and the VIX index as the variable to measure industrial stability to explore the impact of COVID-19 on industrial stability and development and concluded that COVID-19 hurt industrial development. At the same time, Goodell [27] analyzed the impact of COVID-19 on industry, the public, and society by comparing major historical emergencies. According to McKibbin and Fernando [28], the COVID-19 pandemic has reduced the total global economic output by nearly 14%. At the same time, due to the contagion of panic caused by the pandemic, consumers’ hoarding behavior has intensified, resulting in excess demand [29, 30], which in turn has resulted in an imbalance between supply and demand, and thus affected industrial structures. The impact of the novel coronavirus outbreak is introduced into the New Keynesian DSGE model, and a conclusion is drawn that the impact is phased, and the long-term effect is not significant [31]. Some scholars have built a computable general equilibrium model (CGE) to quantitatively analyze the impact of the COVID-19 pandemic and demonstrate the importance of policy effects [32]. Lavopa and Donnelly [33] measure the changing trend of industrial development during the pandemic, and the results showed that after the outbreak of COVID-19, the difficulty of industrial structure transformation increased significantly. Bevilacqua et al. [34] found that the systemic risks of the countries involved in the study increased significantly during the outbreak of COVID-19 from the perspective of global financial risks. COVID-19 has sounded the alarm for the global economy [35]. Ibn-Mohammed et al. [36] conducted research and analysis on the pandemic and showed that its impact on the short-term risks of the industrial real economy was generally controllable. Jiang [37] show that the negative impact of COVID-19 has had obvious effects in the short term, but relatively smooth effects in the long run through the risk growth model. Some scholars have used the factor augmentation vector autoregressive model to investigate the impact of COVID-19 on China’s macro economy and financial market [38]. Other studies have found that COVID-19 has had a huge impact on China’s economy, and production stoppage directly affects the production of enterprises, leading to the reduction of capital utilization and investment of enterprises and a decline in consumer demand [39].

To sum up, the impact of COVID-19 on the industrial economy has been examined from various aspects, including theory and time. However, since the COVID-19 pandemic was a sudden outbreak in late 2019 and early 2020, when looking at regional economic and industrial development in resource-based regions, existing studies have ignored the reduced toughness of industrial structure caused by the disruption of the industrial chain under the impact of COVID-19, and ignored the spatial correlation in factor flow, industrial structures and other aspects of resource-based regions due to their own resource endowment advantages being different from other regions. Moreover, the study of the spatial pattern evolution law of resource-based areas lacks an analysis of the spillover effect. At the same time, studies that consider the impact of COVID-19 mostly focus on the impact on China’s macro economy and industry. Few empirical studies are conducted on resource-based regions alone, and few papers use spatial models. Therefore, this paper studies and analyzes the industrial development trend, the toughness of industrial structure, and the spatial spillover effect of resource-based regions in China under the influence of COVID-19. It provides countermeasures and suggestions for the industrial chain reconstruction and the coordinated development of regional economic resources and the environment in resource-based regions to realize low-carbon, environmental protection, and sustainable development of resources in resource-based regions.

## 3. Research object and data source

China’s resource-based regions are numerous and widely distributed. A total of 262 have been identified in the "National Sustainable Plan of resource-based regions (2013–2020)." According to the general rules of the development of these cities, they can be divided into four types: growth-oriented, mature, recessive, and regenerative [40], based on their development stage, energy security capacity, and sustainable development capacity. Considering the comprehensiveness and representativeness of the study and limited by the availability of data, this paper selects 68 prefecture-level resource-based regions as the research objects nationwide (see Table 1 [41]. The study’s regions cover all types of resource-based regions in the country as far as possible. Among these 68 cities, the growth-oriented ones are mainly distributed in the northeast and west, near the population line of Hu Huanyong [42]. Mature cities account for a large proportion of resource-based regions and are widely distributed, with 60% in the central and western regions. Recessive cities are mainly distributed in the northeast, central, and west. Regenerative cities are mainly distributed in the northeast and central China, with a few in the east and west.

**Table 1:** Development stages and areas of 68 resource-based regions.

Region			Development stage	Region			Development stage	Region			Development stage	Region			Development stage
East				Centre				Northeast				Northeast			
	1	Dongying	Mature		18	Huaibei	Recessive		36	Ansahn	Regenerative		54	Yichun	Recessive
	2	Jining	Mature		19	Huainan	Mature		37	Baotou	Regenerative	Northwest			
	3	Laiwu	Mature		20	Jiaozuo	Recessive		38	Benxi	Mature		55	Baiyin	Recessive
	4	Linyi	Regenerative		21	Jingdezhen	Recessive		39	Chifeng	Mature		56	Baoji	Mature
	5	Longyan	Mature		22	Loudi	Mature		40	Daqing	Mature		57	Jinchang	Mature
	6	Nanping	Mature		23	Luoyang	Regenerative		41	Erdos	Growth-oriented		58	Longnan	Growth-oriented
	7	Sanming	Mature		24	Maanshan	Regenerative		42	Fushun	Recessive		59	Pingliang	Mature
	8	Taian	Mature		25	Nanyang	Regenerative		43	Fuxin	Recessive		60	Qingyang	Growth-oriented
	9	Zaozhuang	Recessive		26	Pingdingshan	Mature		44	Hegang	Recessive		61	Shizuishan	Recessive
	10	Zibo	Regenerative		27	Pingxiang	Recessive		45	Heihe	Mature		62	Tongchuna	Recessive
Centre					28	Puyang	Recessive		46	Hulun Buir	Growth-oriented		63	Weinan	Mature
	11	Bozhou	Mature		29	Sanmenxia	Mature		47	Huludao	Regenerative		64	Wuwei	Growth-oriented
	12	Chenzhou	Mature		30	Shaoyang	Mature		48	Jixi	Mature		65	Xianyang	Growth-oriented
	13	Chizhou	Mature		31	Tongling	Recessive		49	Mudanjiang	Mature		66	Yanan	Growth-oriented
	14	Chuzhou	Mature		32	Xinyu	Recessive		50	Panjin	Regenerative		67	Yulin	Growth-oriented
	15	Ganzhou	Mature		33	Suzhou	Mature		51	Qitaihe	Recessive		68	Zhangye	Regenerative
	16	Hebi	Mature		34	Yicheng	Mature		52	Shuangyashan	Recessive				
	17	Hengyang	Mature		35	Yichun	Mature		53	Wuhai	Recessive				

This paper is based on data collected from these 68 cities, covering 2010 to 2021, with 816 observations [43]. The missing values of individual years are supplemented by linear interpolation. The industrial GDP data used in this paper are from the China Industrial Statistics Yearbook (2010–2021); the scientific expenditure data are from the China Science and Technology Statistics Yearbook (2010–2021); and the year-end employment, fixed asset investment, and the proportion of tertiary industry GDP are from the China Statistical Yearbook (2010–2021) and the statistical yearbook of cities in resource-based regions.

## 4. Methodology

This paper explores the industrial-development rules, industrial-structure evolution characteristics, and spillover effects of resource-based regions in China in the post-pandemic era by firstly selects selecting the Markov chain and the industrial-structure upgrading index to analyze the characteristics of regional industrial-development time-series evolution and industrial-structure toughness. It then builds a spatial Dubin model to analyze the spatial spillover effects of regional industrial development.

### 4.1 Industrial development analysis method

#### 4.1.1 Markov chain

The Markov chain is the method used in this paper to discretize the continuous data in the geographical space and divide it into *n* types [44]. The transfer probability of each spatial type in continuous time is observed, the value being between 0 and 1. The whole state transition process could be performed with n ×. The transition probability of *n* is expressed by *N*_*ij*_*’s* state transition probability matrix. See Table 2 for details.

The probability calculation formula is as follows

$$N_{ij}=\frac{m_{ij}}{m_{i}}$$ (1)

**Table 2:** Markov transition probability matrix.

*t*_*i*_ */t*_*i+*1_	*N*_1_	*…*	*N*_*n*_
*N*_1_	*N*_11_	*…*	*N*_1*n*_
*…*	*…*	*…*	*…*
*N*_*n*_	*N*_*n*1_	*…*	*N*_*nn*_

The probability calculation formula is as follows: *m*_*ij*_ represents the total number of type *i* fields transferred from one region to the next in the whole study period; *m*_*i*_ represents the total number of regions of type *i* in the whole study period. Therefore, *N*_*ij*_ represents the probability of a region from state *i* to state *j*.

#### 4.1.2 Advanced industrial-structure index

The upgrading of the industrial structure refers to the increased added value of products and the optimization of the sector structure. In terms of industrial structure, the main performance is that the ranking of three industrial increases has changed from "one, two, three" to "three, two, one" [45, 46]. The toughness of industrial structures mainly refers to the measurement system of the self-recovery and self-adjustment ability of industrial structures under the impact of sudden public security events. The upgrading of industrial structures is more in line with the significance of this paper’s research on the toughness of industrial structures. Therefore, the upgrading index of industrial structures is selected as the industrial-structure toughness index. The higher the value, the higher the upgrading level of regional industrial structures and the higher the industrial-structure toughness. This study selects the ratio of the output value of the tertiary industry to the output value of the secondary industry to build an index to measure the level of regional industrial upgrading and explore the laws and characteristics of the evolution of industrial-structure toughness.

### 4.2 Spatial spillover measurement model setting

Because there is a strong spatial spillover effect in the industrial development of resource-based regions in China, the level of industrial development of neighboring regions will further affect the regions. So, this paper uses the spatial measurement method to study the spatial spillover effect between neighboring regions and selects the spatial Dubin (SDM) model to comprehensively consider the spillover effect caused by local factors and the spillover effect of neighboring regions on themselves. In the construction of the model, the paper assumes that the production function is in the form of a Cobb–Douglas (C–D) production function that introduces the labor force, capital, and technology of the region, and carries out logarithmic transformation to obtain the general production function form.

lnY=lnA+αlnL+βlnK (2)

as follows: Among them, *α and β* are the parameters to be estimated. *lnY*, *lnA*, *lnL*, and *lnK* are the logarithmic forms of regional industrial development, technology, capital, and labor, respectively. When the introduce the industrial structure index [47] in introduced, the spatial panel regression model of the C–D production function is constructed.


$$lnY_{it}=\alpha +\lambda W_{it}lnY_{it}+\beta_{1}lnX_{1it}+\beta_{2}lnX_{2it}+\beta_{3}lnX_{3it}n+\beta_{4}lnX_{4it}+\delta W_{it}lnX_{it}+\varepsilon_{it}$$ (3)

The model setting form is as follows: Where *i* is the city, *t* is the time, *Y* is the industrial development, and *X*_1_, *X*_2_, and *X*_3_, respectively, represent the regional labor force, capital, and technology factors; *X*_4_ is the regional industrial structure. *β*_1_, *β*_2_, *β*_3_, and *β*_4_, respectively, represent the influence coefficient of each variable of region *i* on the industrial development of the region; *δ* is the coefficient matrix; *ε*_*it*_ is a random perturbation term.

### 4.3 Indicator construction

Based on the C–D production function, this paper introduces the factors of labor, capital, and technology input and analyzes the interrelation and influence factors of industrial development and economic development among regions. The explained variable uses the industrial GDP (*Y*) of each region as the evaluation index of the overall development level of the resource-based region industry. The explained variable uses the local year-end employment (*X*_1_), fixed asset investment (*X*_2_), and scientific expenditure (*X*_3_) as the evaluation index of labor, capital, and technology input factors. In addition, considering the practical reasons, the proportion of GDP of the tertiary industry (*X*_4_) is introduced into the model to indicate the change in regional industrial structure. Table 3 shows the symbols, names, and meanings of each variable.

**Table 3:** Variable names and meanings.

Character	Name	Meaning
Y	Industrial GDP (10000 yuan)	Measure regional industrial development level
X_1_	Employment at the end of the year (10000)	Measure labor input
X_2_	Fixed asset investment (10000 yuan)	Measuring capital investment
X_3_	Scientific expenses (10000 yuan)	Measure technology investment
X_4_	Proportion of tertiary industry GDP (%)	Measure the change of regional industrial structure

## 5. Empirical results

### 5.1 Timing characteristics of industrial development

This paper uses the Markov chain to judge the stability of the industrial-development level in China’s resource-based regions and the characteristics of the time-series evolution of each region. At the same time and due to the particularity of the industries in resource-based regions, when measuring the industrial-development level, the industrial GDP is selected as the indicator, taking the average of industrial GDP as the benchmark: 75%, 100%, and 125% as the cut-off points. The industrial development level of 68 resource-based regions is divided into four levels. See Table 4 for details.

**Table 4:** Industrial development level.

State	Industrial development level	Grading standard
N_1_	Low	$x\leq 0.75\bar{x}$
N_2_	Medium-low	$0.75\bar{x}<x\leq \bar{x}$
N_3_	Medium-high	$\bar{x}<x\leq 1.25\bar{x}$
N_4_	High	$x>1.25\bar{x}$

Considering the impact of COVID-19 on the development of resource-based regions in China, the whole research period is divided into two stages—namely, 2010–2019 and 2020–2021—and the Markov transfer probability matrix of the industrial-development level is calculated, as shown in Table 5. The evolutionary characteristics of China’s regional industrial-development level from 2010 to 2021 are then analyzed.

**Table 5:** Markov transfer of 68 cities.

Time	Classification	N_1_	N_2_	N_3_	N_4_
2010–2021	N_1_	0.860	0.140	0	0
	N_2_	0.011	0.693	0.296	0
	N_3_	0	0.034	0.753	0.213
	N_4_	0	0	0.041	0.959
2010–2019	N_1_	0.852	0.148	0	0
	N_2_	0.007	0.674	0.319	0
	N_3_	0	0.037	0.741	0.222
	N_4_	0	0	0.024	0.976
2020–2021	N_1_	0.893	0.107	0	0
	N_2_	0.057	0.745	0.198	0
	N_3_	0	0.053	0.765	0.182
	N_4_	0	0	0.088	0.912

The results show that, 1. There are four levels of regional industrial development: low, medium-low, medium-high, and high, and the level of industrial development in most regions is low. The diagonal values of each matrix in Table 5 represent the probability that the industrial-development level remains unchanged. It can be seen that the diagonal value is greater than the non-diagonal value, with a maximum value of 0.976 and a minimum value of 0.674. It shows that most regions tend to maintain the original industrial development type. From the matrix, it can also be seen that the probability of occurrence of low and high levels on the diagonal is relatively high, indicating that the low- and high-level types have strong stability, the probability of occurrence of moderately low and medium to high levels is relatively small, and the type of stability is slightly weak.

2. From 2010 to 2019, the probability of a downward transfer of grades is generally lower than that of an upward transfer, which indicates that the overall industrial development is good. Among the types of upward transfer, the probability of a downward transfer of low, medium-low, and medium high levels are 0.148, 0.319, and 0.222, respectively, indicating differences in the probability of regional migration between the different levels. Among them, the probability of a downward transfer of low levels is small, which may be due to the weak economic foundation of these regions. The industrial development is still at a lower level, and the construction speed is slow. The probability of an upward migration of low- and medium-high regions is slightly higher, mainly due to the relatively mature industrial system. However, in general, the transfer probability between grades is low, which means that the probability of China’s resource-based regions achieving industrial leapfrog development in the short term is low.

3. The diagonal value from 2020 to 2021 has changed from the previous stage, and the overall value is larger than the previous stage, indicating that the probability of the industry development level being unchanged in this stage is increasing. This means that after COVID-19, the industry development level of resource-based regions in China tended to be more stable. In addition, compared with 2010–2019, the values on both sides of the diagonal from 2020 to 2021 significantly decreased, indicating that the probability of upward and downward transfer has decreased. Although the downward-transfer probability at this stage is still generally less than the upward-transfer probability, the amplitude is far less than in the previous stage, indicating that under the impact of COVID-19, the industrial development of resource-based regions in China has a trend of maintaining the original state and stagnating.

4. In general, the industrial development level in different regions has increased steadily during the study period, but it can be clearly seen that after the impact of COVID-19, industrial development in China’s resource-based regions tended to be more stable, and the development speed was far less than the previous stage. The reason may be that the "closure" brought about by the "shutdown" of industry led to the slow development of industry and even tended toward maintaining the original state. In addition, the value on the non-diagonal line represents the probability of change between different industrial development levels. The maximum value on the non-diagonal line is 0.319, which is far lower than the value on the diagonal line. The law of the value is that the diagonal line is used as the dividing line to gradually reduce the upper right and lower left, indicating that even if there is an increase or decrease in the industrial development level, it is an "adjacent transfer," and the probability of leapfrog change in the industrial development level is low.

### 5.2 Advanced level

The industrial-structure upgrading index is used to express the upgrading degree of the industrial-structure level in 68 cities to analyze the evolution of regional industrial-structure toughness. Three time points are selected: 2010, 2019, and 2021; the Z-core method is used to pre-process the industrial-structure upgrading index [48]. Because the geometric interval classification method [49] helps ensure the same number of samples of all types and integrates the advantages of the natural breakpoint method and the quantile method, this method is used to divide the advanced level of the industrial structure of resource-based areas into five gradients: low-low, low, medium, high, and high-high, as shown in Table 6.

**Table 6:** Advanced level of industrial structure among regions.

Region		2010	2019	2021	Region		2010	2019	2021
Dongying	1	High	High	High	Yichun	35	Low	High	Medium
Jining	2	Medium	High	High-high	Ansahn	36	Low	High	Medium
Laiwu	3	High	High	Medium	Baotou	37	High-high	High-high	High-high
Linyi	4	High	High-high	High-high	Benxi	38	Low	Medium	Medium
Longyan	5	Medium	High	High	Chifeng	39	Low	Medium	Medium
Nanping	6	Low	Low	Low	Daqing	40	Medium	Medium	Medium
Sanming	7	Low	Low	Low	Erdos	41	High-high	High	High
Taian	8	High	Medium	High	Fushun	42	Low-low	Low	Medium
Zaozhuang	9	High	High	Medium	Fuxin	43	Medium	Medium	High
Zibo	10	High-high	High-high	High-high	Hegang	44	Low-low	Low-low	Low-low
Bozhou	11	Low-low	High	High-high	Heihe	45	Low-low	Low-low	Low-low
Chenzhou	12	Medium	High-high	High	Hulun Buir	46	Low	Medium	Low
Chizhou	13	Medium	High	High-high	Huludao	47	Low	Medium	Medium
Chuzhou	14	Low	High	High	Jixi	48	Low-low	Low-low	Low-low
Ganzhou	15	Low	High	High-high	Mudanjiang	49	High	High	High-high
Hebi	16	Low	Medium	High	Panjin	50	Low-low	Low-low	Low-low
Hengyang	17	Low	High-high	High	Qitaihe	51	Medium	Medium	High
Huaibei	18	Medium	High-high	High-high	Shuangyashan	52	Low-low	Low-low	Low-low
Huainan	19	Medium	High	High-high	Wuhai	53	Medium	High	High
Jiaozuo	20	Medium	High	High-high	Yichun	54	Low-low	Low	Low-low
Jingdezhen	21	High	High-high	High-high	Baiyin	55	Medium	Low	Low
Loudi	22	Medium	High	Medium	Baoji	56	Medium	Medium	Medium
Luoyang	23	High	High-high	High-high	Jinchang	57	Medium	Medium	Medium
Maanshan	24	High	High-high	High	Longnan	58	Low	High	High
Nanyang	25	Low	High	High	Pingliang	59	Low	Medium	Medium
Pingdingshan	26	Medium	High	High-high	Qingyang	60	Low	Medium	Medium
Pingxiang	27	Medium	High-high	High-high	Shizuishan	61	High	High	High-high
Puyang	28	Low	High	High	Tongchuna	62	High	High-high	High-high
Sanmenxia	29	Medium	High	High	Weinan	63	Medium	Low	Low
Shaoyang	30	Low	High	High-high	Wuwei	64	Low-low	Medium	High
Tongling	31	High	High-high	High-high	Xianyang	65	Low	Low	Low
Xinyu	32	High	High-high	High-high	Yanan	66	Medium	Medium	Low
Suzhou	33	Low-low	High	High-high	Yulin	67	High	Medium	Medium
Yicheng	34	Medium	High	High	Zhangye	68	Low-low	Medium	Medium

Table 6 shows:

1. Significant differences in 2010 in the level of industrial-structure upgrading among regions, and the degree of industrial-structure toughness is uneven; there is no significant trend of convergence. Among the studied regions, only three have a high level of regional industrial-structure upgrading (namely, Zibo, Baotou, and Ordos), which shows that these three regions have a high degree of industrial-structure toughness and a strong ability to cope with external emergencies. However, there are 30 regions with low-low and low industrial structures, which means that the industrial-structure toughness in these regions is significantly lower than in other regions. The main causes may be that these regions are rich in resources and are located in remote areas, the secondary industry accounts for a large proportion, the population at the same time is relatively small, and the impact of public emergencies on the industrial structure in these regions is small.

2. From 2011 to 2019, the industrial-structure upgrading level in various regions greatly improved, as did the industrial-development trend in most regions. The number of regions at the top level increased from 3 to 13, of which Hengyang directly stepped into the top level from the low level in 2010, and Chenzhou, Huaibei, and Pingxiang stepped into the top level from the middle level, realizing leapfrog development. This shows that these cities developed rapidly at this stage, with obvious industrial-structure optimization and substantial improvement of industrial-structure toughness. The number of low-low-grade and low-grade regions decreased to 12, including Nanping, Sanming, Hegang, Heihe, Jixi, Panjin, Shuangyashan, and Xianyang. The grades of Fushun and Yichun have not changed. Fushun and Yichun have withdrawn from the lower-grade sequence, which indicates that these regions are accelerating the upgrading of industrial-structure adjustment and occasionally can speed up, and the industrial-structure toughness has gradually improved.

3. Under the impact of COVID-19 from 2020 to 2021, the level of industrial-structure upgrading in each region tended to be more stable. Except for retrogression in some regions, the level of upgrading in other regions continued to improve steadily. The number of regions with high and high-high levels of industrial-structure upgrading increased to 39, more than half of the total sample, and the only regions with lower levels were Hegang, Heihe, Jixi, Panjin, Shuangyashan, and Yichun, of which only Yichun experienced a regression from a low level to a low-low level. In 2021, of the 17 regions where the industrial structure could have been upgraded, none was upgraded, 40 regions remained unchanged, and 11 regions were downgraded. This shows that after entering the post-pandemic era, China’s resource-based regions may have suffered from industrial-chain fractures and reduced industrial-structure toughness, but regions with high industrial-structure toughness could still upgrade or maintain the original level and have a strong resistance to the impact of COVID-19.

4. In general, although there was regional heterogeneity in the evolution of the advanced industrial structure from 2010 to 2021, it has faced the impact of COVID-19 since 2020. Most regions show an upward trend as they evolve and adjust to the "three, two, one" industrial structure. Among them, Dongying, Linyi, Jingdezhen, Luoyang, and Baotou have always been at a higher level of industrial-structure upgrading, and their industrial-structure toughness is stable, which shows that these regions have a solid economic foundation, a mature industrial system, and strong resistance to emergencies. These factors can promote the upgrading of the industrial structure. However, the industrial-structure upgrading level in Hegang, Heihe, Jixi, Panjin, and Shuangyashan has always been low, indicating that the industrial-structure toughness in these regions has always been low, and the resistance to external emergencies is low. It is necessary to continue to accelerate industrial-structure adjustment. On the whole, the numbers of medium-grade, high-high-grade, and high-grade regions have steadily increased, the overall level of industrial-structure upgrading has steadily improved, and the toughness of industrial structures has steadily improved.

### 5.3 Spatial spillover effects

#### 5.3.1 Moran’s index

This paper uses Moran’s index to test whether the industrial development of each region presents spatial autocorrelation. It analyzes the spatial correlation of industrial development.


$$Moran\prime sI=\frac{\sum_{i=1}^{n}\sum_{j=1}^{n}w_{ij}(Y_{i}-\overset{-}{Y})(Y_{j}-\overset{-}{Y})}{S^{2}\sum_{i-1}^{n}\sum_{j=1}^{n}w_{ij}}$$ (4)

The specific calculation formula is as follows: In formula (4), *S*^2^ is the sample variance, $\bar{Y}$ represents the sample mean; *Y*_*i*_ and *Y*_*j*_ represent the observed values of the *ith* and *jth* regions; and *w*_*ij*_ is the spatial weight matrix. Table 7 shows the global Moran’s I index and its statistical test results. The urban-rural income gap between 2010 and 2021 can be seen in the table.

**Table 7:** Global Moran’s I index of the urban-rural income gap from 2010 to 2021.

Variables	I	E(I)	sd(I)	z	p-value*
lnY2010	0.149	-0.017	0.029	5.711	0.000
lnY2011	0.150	-0.017	0.029	5.768	0.000
lnY2012	0.144	-0.017	0.029	5.562	0.000
lnY2013	0.166	-0.017	0.029	6.316	0.000
lnY2014	0.201	-0.017	0.029	7.522	0.000
lnY2015	0.226	-0.017	0.029	8.344	0.000
lnY2016	0.231	-0.017	0.029	8.512	0.000
lnY2017	0.234	-0.017	0.029	8.632	0.000
lnY2018	0.237	-0.017	0.029	8.738	0.000
lnY2019	0.238	-0.017	0.029	8.775	0.000
lnY2020	0.238	-0.017	0.029	8.772	0.000
lnY2021	0.238	-0.017	0.029	8.772	0.000

Moran’s index in Table 7 is calculated using the spatial geography matrix. It can be seen that the global Moran’s I index value of industrial development from 2010 to 2021 is significantly positive at a significance level of 1%, indicating that the change in the industrial-development level in each region has a significant positive global spatial correlation—that is, the industrial-development level of adjacent regions is close. In addition, the overall Moran’s I index value of the urban-rural income gap between 2010 and 2021 shows a gradual upward trend, indicating that the spatial dependence of the industrial-development level in various regions is gradually becoming stronger, which is in line with the development trend of industrial integration.

#### 5.3.2 Model correlation test

The LM test, LR test (Tables 8 and 9), and Wald test were carried out before the spatial spillover test. The results reject the original hypothesis at the level of 1%, indicating that it cannot be simplified into the spatial panel error model and the spatial panel lag model, so the selected model is the spatial Dubin model. Secondly, the Hausman test (Table 10) shows that the original hypothesis is rejected at a significance level of 1%—that is, the random effect is rejected, so this paper selects the spatial Dubin fixed effect model.

**Table 8:** LM test.

	LM test
LM-lag	25.287***
R-LM-lag	15.772***
LM-error	9.792***
R-LM-error	3.995***

**Table 9:** LR test.

	LR test
SAR	21.9***
SEM	29.9***

**Table 10:** Hausman test.

	Hausman test
Statistics	97.06***

#### 5.3.3 Spatial Dubin model

This paper uses the spatial Dubin model for regression, and the specific regression results are shown in Table 11.

**Table 11:** Regression results of spatial Dubin model.

	(1)	(2)	(3)
	Individual fixation	Time fixation	Double fixation
lnX_1_	0.1872***	0.2945***	0.1848***
	(4.87)	(5.39)	(4.87)
lnX_2_	0.4345***	0.7471***	0.4068***
	(12.93)	(15.60)	(11.85)
lnX_3_	0.0633***	0.0804**	0.0560***
	(3.92)	(2.20)	(3.50)
lnX_4_	-0.3075***	-1.0195***	-0.3151***
	(-4.20)	(-9.13)	(-4.38)
WlnX_1_	0.0373	1.5894***	0.2518
	(0.36)	(6.42)	(1.28)
WlnX_2_	-0.0900	-0.8500***	0.2745**
	(-0.85)	(-5.13)	(2.04)
WlnX_3_	0.1258**	0.7961***	0.2166***
	(2.05)	(5.39)	(2.87)
WlnX_4_	-0.0111	4.5846***	0.9570**
	(-0.07)	(5.91)	(2.54)
R^2^	0.6415	0.6826	0.6552

Tip:*,**and***represent significance levels of 10%, 5%, and 1%, respectively. Standard errors are in parentheses.

It can be seen from the results shown in Table 11 that model (1) (2) (3) represents the spatial Dubin regression results of individual fixed, time fixed, and individual and time two-way fixed, respectively. Among the three fixed spatial Dubin models, most of the variables are significant, and the time-fixed model has the highest goodness of fit. Therefore, this paper selects the spatial Dubin model with fixed time effects for analysis. That is the model in Table 11 (2).

First, labor input has a significant positive impact on the level of industrial development, with a coefficient of 0.2945 and significance at the level of 1%, indicating that the input of labor factors can promote the level of industrial development—that is, the more labor input, the better the level of industrial development. Secondly, capital also plays an important role in the process of industrial development. Capital has a positive impact on the level of industrial development at a significance level of 1%, with a coefficient of 0.7471, indicating that capital can also promote industrial development, and its coefficient is greater than the coefficient of labor input of 0.2945, indicating that the impact of capital on industrial development is greater than that of labor. Thirdly, technology has a positive impact on the level of industrial development at a significance level of 5%, with a coefficient of 0.0804. Compared with labor and capital factors, technology has a slightly weaker impact on the level of industrial development, but it can be seen that labor, capital, and technology can have a positive impact on the level of industrial development. Finally, a proportion of the tertiary industry has a significant negative impact on the development level of the industry, with a coefficient of -1.0195, which indicates that the development of the tertiary industry has squeezed the development space of other industries, resulting in the poorly coordinated development of various industries, so it has a negative impact.

According to the spatial spillover effect of the industrial-development level, the input of labor-production factors has a significant positive effect on the level of industrial development in neighboring regions, with a coefficient of 1.5894, indicating that the cross-regional flow of labor has a good impact on industrial development. The spillover effect of capital investment is negative, with a coefficient of -0.85. This is because although the flow of capital will promote the industrial development of the inflow area, it will hinder the industrial development of the outflow area. The spillover effect of technological factors on the level of industrial development is significantly positive, with an impact coefficient of 0.7961, indicating that technological development not only promotes the upgrading of industrial structures in the region but also radiates to the surrounding areas. The spillover coefficient of the proportion of the tertiary industry is significantly positive, with a value of 4.5864, indicating that the optimization of the industrial structure has obvious positive externalities and drives the comprehensive development level of the surrounding industries.

### 5.4 Robustness check

To further test the robustness of the previous regression results, this paper replaces the geo-spatial weight matrix with the economic distance spatial weight matrix to test the spatial Dubin model. The specific results are shown in Table 12. From the regression results, the spatial Dubin model with fixed time has the best fitting effect, followed by the individual fixed, and finally the spatial Dubin model with two-way fixed. The spatial Dubin model with fixed time effects is used for analysis. Labor force, capital, and technology have a significant positive relationship with the level of industrial development, with coefficients of 0.1632, 0.5501, and 0.0774, respectively. This shows that capital has the greatest impact on the level of industrial development among the above three factors. At the same time, the proportion of the tertiary industry’s GDP reduces the level of industrial development. This is because the development of the regional tertiary industry puts forward certain requirements on local resources and the environment. Vigorously developing the tertiary industry will inhibit the comprehensive development of the local industry in the short term, but because of its positive externalities, it can promote the industrial development of the neighboring areas to a certain extent. Table 12 shows that, after replacing the weight matrix, the empirical results in the previous article remain stable.

**Table 12:** Results of robustness test.

	(1)	(2)	(3)
	Individual fixation	Time fixation	Double fixation
lnX_1_	0.1944***	0.1632***	0.1981***
	(5.22)	(2.92)	(5.19)
lnX_2_	0.4743***	0.5501***	0.4708***
	(14.12)	(10.82)	(14.01)
lnX_3_	0.0734***	0.0774**	0.0680***
	(4.53)	(2.25)	(4.21)
lnX_4_	-0.2526***	-0.9576***	-0.2577***
	(-3.68)	(-8.61)	(-3.54)
WlnX_1_	0.0410	0.6588***	0.0736
	(0.66)	(7.50)	(1.02)
WlnX_2_	-0.0670	-0.1282*	-0.0396
	(-1.37)	(-1.91)	(-0.78)
WlnX_3_	0.0421	0.3029***	0.0248
	(1.61)	(6.18)	(0.91)
WlnX_4_	0.0843	0.8267***	0.0444
	(1.02)	(4.21)	(0.41)
R^2^	0.7593	0.8087	0.7589

Tip: *, ** and *** represent significance levels of 10%, 5%, and 1%, respectively. Standard errors are in parentheses.

## 6. Conclusions

This paper took 68 resource-based prefecture-level cities in China from 2010 to 2021 as the research object. It then comprehensively used the Markov chain, the industrial structure upgrading index, and the spatial econometric model using the COVID-19 outbreak as a turning point, to analyze the industrial development evolution, industrial-structure toughness change, and spatial spillover effect of these resource-based regions in China. The main conclusions are as follows.

1. Most regions tend to maintain the original type of industrial development, and the overall stability of industrial development is slightly weak. The improvement or reduction of the development level of different levels in the region is "adjacent transfer," and the probability of leapfrog change in the industrial-development level is low.

2. The evolution of industrial-structure upgrading is significantly heterogeneous among regions. Due to the different resource conditions of each region, the industrial-structure toughness is different. After the impact of COVID-19, the regional industrial structure above the middle level had better toughness and resistance. Therefore, they basically maintained the original level or rose slowly and steadily. However, in regions where the original level of industrial-structure upgrading was relatively low, the industrial-structure toughness itself was poor, and some regions regressed.

3. There was a significant spatial spillover effect in the development of regional industries. While the input of labor and technological factors promoted the development of local industries, its spillover effect also led to the improvement of the level of industrial development in the region. The development of the tertiary industry may squeeze the space for industrial development and, to some extent, inhibit the development of local industries. Capital investment will promote the region’s industrial development but hinder the industrial development of the outflow region.

## 7. Suggestions

Baesd on existing research, this paper examined the industrial development, industrial-structure toughness, and spillover effects of resource-based areas in China in the context of the post-pandemic era. The conclusions are of practical significance. Due to the impact of COVID-19, the development situation is not optimistic, so this article puts forward the following three suggestions.

Further optimize and adjust the industrial structure. There are different types of resource-based regions in China. Some are mainly oil and gas, some are mainly mineral resources, and some are mainly ecological resources. Due to the different types of cities with different resources, the direction of industrial-structure optimization is diverse. After entering the post-pandemic era, the rupture of the industrial chain and supply chains point out the direction of future research. In order to achieve low-carbon, environmental protection, and sustainable development of resources, we should consider the impact of the reduced resilience of industrial structures brought about by COVID-19. The toughness can be improved by: ① increasing the contribution of resources and expanding supply; ② improving resource allocation; ③ realizing the scientific development of the industry. At the same time, the heterogeneity index can be used to carry out the differential evaluation of the industrial development of resource-based regions in China and select different industrial-structure adjustment directions according to the different resource endowments and characteristics of different cities.

Further study the driving mechanism of industrial development. China’s resource-based regions have a wide variety of resources of differing quality, but their industrial chain is short, and the value chain is at the low end. They are the most complex areas for China’s long-term green economic development and the key areas for China’s sustainable development. The industrial development of resource-based regions in China is not only affected by the input of the three basic elements of labor, capital, and technology but also by the resource-guarantee capacity and environmental carrying capacity. Future research can comprehensively analyze the driving mechanism of industrial development based on the characteristics of different resource-based regions and incorporate more regional characteristic variables into the empirical model for further analysis.

Fuether explore the heterogeneity of industrial development. Due to the differences in resource dependence, pollution emissions, and policy preferences across the country, the elasticity coefficient of factor input and industrial development in different industries may be completely different. In addition, when selecting research objects, this paper tried to select resource-based regions equally distributed in the central, western, eastern, and northeastern parts of the country. According to the classification of national resource-based regions in the "National Sustainable Plan of resource-based regions (2013–2020)," the research objects involved are divided into four categories: growth-oriented, mature, recessive, and regenerative. Resource-based regions in different growth cycles have different resource-guarantee capabilities and social sustainable development capabilities. Future research can consider the heterogeneity impact of industries and growth cycles according to the types identified in this paper and, at the same time, conduct more in-depth classification and comparative analysis to verify the heterogeneity impact of industries, growth cycles, and other factors on the industrial development of resource-based regions in China, and analyze the causes.

## Supporting information

S1 Raw data (ZIP)
